# Vitamin D levels and human sperm DNA fragmentation: a prospective, cohort study

**DOI:** 10.1186/s12610-022-00166-8

**Published:** 2022-09-13

**Authors:** Elise Blaseg, Tiffany Von Wald, Keith A. Hansen

**Affiliations:** 1 Medical Student, University of South Dakota Sanford School of Medicine, Vermillion, SD USA; 2 Sanford Health Fertility and Reproductive Medicine, Sanford Women’s, Sioux Falls, SD USA; 3 Department of Obstetrics and Gynecology, University of South Dakota Sanford School of Medicine, Sioux Falls, SD 57105 USA

**Keywords:** Sperm chromatin structure assay, Sperm DNA fragmentation %, Vitamin D, Male infertility, Hypovitaminosis D

## Abstract

**Background:**

Intracytoplasmic sperm injection (ICSI) has revolutionized the treatment of couples with male factor infertility but results remain suboptimal and suggest the need for further investigation into the molecular biology of spermatozoa. Vitamin D has been implicated in spermatogenesis and sperm function. Hypovitaminosis D has been associated with abnormal testicular function, including elevated sperm DNA fragmentation in a murine model. This study’s objective was to evaluate if there is a correlation between Vitamin D sufficiency and human spermatozoa DNA fragmentation index % (DFI%) in infertile couples.

**Results:**

A prospective cohort study using a consecutive, convenience sample of subjects with infertility. The primary endpoint was the effect of Vitamin D sufficiency on human spermatozoa DFI%, and secondary outcomes included Vitamin D’s effect on moderate DFI%, high DFI%, High DNA stainability % (HDS%), sperm density (million/mL), sperm total motility (% total) and sperm strict morphology (% total). Of the 111 participating, 9 were excluded, leaving 102subjects. The subjects were stratified by vitamin D levels: deficient (< 20 ng/mL; *n* = 24), insufficient (20–30 ng/mL; *n* = 43),, and sufficient (> 30 ng/mL; *n *= 35). There were no statistical difference between the categories of serum vitamin D levels and sperm DFI% as well as the secondary outcomes. An increased BMI was associated with low serum vitamin D levels (*p* = 0.0012).

**Conclusion:**

Vitamin D deficiency was not associated with sperm DFI% or routine sperm parameters. Previous animal and human studies have demonstrated conflicting results between sperm parameters and Vitamin D levels. Redundant pathways in Vitamin D and calcium homeostasis in the human male reproductive tract may maintain essential reproductive processes during Vitamin D insufficiency or deficiency.

**Trial registration:**

Trial Registration Number: MOD00002311 (ClinicalTrials.gov).

## Résumé

**Contexte:**

L’injection intracytoplasmique de spermatozoïdes (ICSI) a révolutionné le traitement des couples avec infertilité masculine, mais les résultats restent sous-optimaux et suggèrent la nécessité d’aller plus loin dans l’étude de la biologie moléculaire des spermatozoïdes. La vitamine D a été impliquée dans la spermatogenèse et les fonctions des spermatozoïdes. Dans un modèle murin, l’hypovitaminose D a été associée à une fonction testiculaire anormale, y compris une fragmentation élevée de l’ADN des spermatozoïdes. L’objectif de cette étude était d’évaluer s’il existait une corrélation entre un taux suffisant en vitamine D et l’indice de fragmentation de l’ADN (DFI) des spermatozoïdes humains chez les couples infertiles.

**Résultats:**

Une étude de cohorte prospective utilisant un échantillon consécutif et approprié de sujets atteints d’infertilité. Le critère d’évaluation principal était l’effet d’un taux suffisant en vitamine D sur le DFI des spermatozoïdes humains; et les critères de jugement secondaires comprenaient l’effet de la vitamine D sur un DFI modéré, un DFI élevé, un taux élevé de coloration de l’ADN (HDS), le nombre des spermatozoïdes (millions/mL), la motilité totale des spermatozoïdes et sur la morphologie stricte des spermatozoïdes. Sur les 111 participants, 9 ont été exclus, laissant 102 sujets. Les sujets ont été stratifiés par niveau de vitamine D : déficient (<20 ng/mL ; *n*= 24), insuffisant (20-30 ng/mL ; *n*=43) et suffisant (>30 ng/mL ; *n*=35). Il n’y a eu aucune différence statistique entre les catégories de taux sériques de vitamine D et le pourcentage de DFI de spermatozoïdes ainsi qu’avec les critères de jugement secondaires. Une augmentation de l’indice de masse corporelle était associée à de faibles taux sériques de vitamine D (*p* = 0,0012).

**Conclusion:**

La carence en vitamine D n’est pas associée au pourcentage l’indice de fragmentation de l’ADN de spermatozoïdes ou aux paramètres de routine des spermatozoïdes. Des études antérieures chez les animaux et les humains ont montré des résultats contradictoires entre les paramètres des spermatozoïdes et les niveaux de vitamine D. Les voies redondantes dans l’homéostasie de la vitamine D et du calcium dans l’appareil reproducteur masculin humain peuvent maintenir les processus essentiels à la reproduction lors d’insuffisance ou de carence en vitamine D.

**Mots-clés:**

Test de structure de la chromatine des spermatozoïdes, fragmentation de l’ADN des spermatozoïdes, vitamine D, infertilité masculine, hypovitaminose D.

## Introduction

Idiopathic male factor infertility is common and defined as abnormal semen parameters with no determined underlying etiology [1]. Current treatments for unexplained male factor are often empiric, based on tenuous data at best [2]. The development of intracytoplasmic sperm injection (ICSI) has allowed infertile males to become biologic fathers. However, in vitro fertilization (IVF)with ICSI is a complex, expensive procedure in which healthy women undergo ovulation induction and surgical retrieval of oocytes with attendant risks, highlighting the importance of continued investigation into potential causes and treatments of idiopathic male factor [3]. Improvements in our understanding of spermatozoa molecular biology may improve treatments and successful conception through natural or assisted reproduction.

The semen analysis has been the gold standard for determining male fertility [4]. While important in the evaluation of the male, a semen analysis by itself cannot assess the capacity of the sperm to fertilize an ova and does not give a complete understanding of the health and function of the male reproductive organs [5]. One area of investigation that shows promise in evaluating male subfertility is sperm DNA fragmentation tests including the TUNEL assay, the Comet assay, the Sperm Chromatin Dispersion test, and the Sperm Chromatin Structure Assay (SCSA) [6–8]. The Sperm Chromatin Structure assay (SCSA), is a precise and repeatable flow cytometry analysis, which is used to measure acid-induced DNA fragmentation. The primary benefit of the SCSA is when the DNA Fragmentation Index % (DFI%) is > 25% IVF with ICSI is more likely to be successful than intrauterine insemination. An elevated DFI% also allows the patient the opportunity to make lifestyle changes and consider medical therapies to improve the value. [9].Vitamin D has an established role in calcium metabolism and skeletal health,as well as pleiotropic genomic and non-genomic effects in various organs throughout the body, including the reproductive system [10]. The Vitamin D Receptor (VDR) and Vitamin D metabolizing enzymes are found throughout the male reproductive tract, including testicles, prostate, seminal vesicles, epididymis, Leydig cells and even the spermatozoa, which has resulted in increased interest on the role of Vitamin D in male fertility [11, 12]. A study in Denmark found samples with more than two thirds of the sperm staining positive for the Vitamin D inactivating enzyme, CYP24A1 had four times greater likelihood of achieving pregnancy with intrauterine insemination [11]. A 2020 review found that vitamin D may have a positive effect on fertility potential, possibly due to an impact on sperm motility [13]. However, animal and human studies on Vitamin D’s effect on the male reproductive system have been inconsistent [13–16].

The purpose of this study was to evaluate Vitamin D sufficiency on human spermatozoa DNA fragmentation % (DFI%). Secondary endpoints included effect of Vitamin D levels on High DNA Stainability % (HDS%) and other sperm parameters including density (million/mL), total motility (% total), and morphology(% total).

## Materials and methods

This was a convenience sample with consecutive recruitment of subjects, who presented to a Midwestern mid-size Reproductive Endocrinology and Infertility Clinic for infertility; both primary or secondary between March 26,2019 and April 1, 2020. The inclusion criteria were male patients between 21 and 55 who presented with a history of infertility (attempted pregnancy for greater than or equal to one year, or greater than or equal to six months if the female partner was over 35 years of age).. Exclusion criteria were severe oligozoospermia (≤ 1 million/mL) in which there were inadequate numbers of spermatozoa to perform the SCSA. All subjects signed a written consent for participation. Our primary outcome was the Vitamin D level and Total DFI% measured with the SCSA®. Secondary outcomes included moderate DFI%, High DFI% and High DNA Stainability %(HDS%), sperm density (million/mL), total motility (% of total), and strict morphology(% of total). When gated the raw SCSA data can be separated into normal, moderate and high DFI% [9]. Previous studies evaluating sperm morphology after cell sorting, demonstrate that normal and moderate DFI% have normal morphology, while high DFI% have elongated nuclei and signs of apoptosis. Currently, Total DFI% is usually reported, but in the future with advances in cell sorting and ICSI one may be able to select sperm with moderate DFI% and improve outcomes [8, 9].

The demographic and laboratory data was extracted from the patients’ electronic medical records (EMR) (Epic Systems Corporation, Verona, Wisconsin) and independently recorded by 2 investigators (eb,kh) onto a piloted data sheet. Also the meteorological season of the year when the blood sample for Vitamin D was obtained was recorded: Spring-March 1 to May 31, Summer-June 1 to August 31, Fall-September 1 to November 30, and Winter-December 1 to February 28. The two investigators independently entered patient data from the data sheet into separate, de-identified spreadsheet files using Microsoft Excel. After completion, the two Excel spreadsheet files were electronically compared and identified differences were resolved by comparison to original data in the EMR. Body Mass Index (BMI) was calculated from weight (in kilograms) and height (in meters squared) using the NIH BMI calculator (BMI = kg/M^2^).

Semen samples were obtained by masturbation after 2–4 days of abstinence. The samples were ejaculated into a nontoxic specimen container and placed in a 37 °C water bath for 20–30 min to allow for liquefaction. The semen analysis was performed manually by two trained technicians in the Andrology laboratory after liquefaction using the fifth edition of the World Health Organization (WHO) guidelines. The normal semen parameters included semen volume of ≥ 1.5 mL, sperm density of ≥ 15 million/mL, total sperm motility of ≥ 40%, and morphology of ≥ 4% using Kruger’s strict criteria [17]. Oligozoospermia was defined as < 15 Million sperm per mL, asthenozoospermia was defined as a total sperm motility of < 40%, and teratozoospermia defined as < 4% normal forms by strict morphology.

A 0.2–0.5 mL sample of the raw semen was placed in a 1 mL cryovial and snap frozen in liquid nitrogen, which was then shipped to SCSA® Diagnostics for assay. The Sperm Chromatin Structure assay (SCSA) which is a highly precise and repeatable, flow cytometry analysis was used to measure acid-induced DNA fragmentation. The semen sample is treated with acidic buffer solution (pH = 1.2) to allow the DNA to open at sites of DNA fragmentation and then treated with Acridine Orange (AO) staining solution composed of 0.20 M Na_2_HPO_4_, 0.1 M citric acid buffer (pH 6.0), 1 mM EDTA, 0.15 M NaCl, and 6.0 ug/mL chromatographically purified AO. After AO treatment the sample was run through the flow cytometer where it is exposed to a 488 nm wavelength excitation beam from a 15–35 mW laser. Red (630–650 nm) and green (515–530 nm) filters collect the fluorescent signal from the excited, AO stained sperm cells. AO is a metachromatic dye that fluoresces green when associated with native, double-stranded DNA and red when associated with single-stranded DNA. An increase in red/green fluorescence is consistent with increased DNA fragmentation. Parameters are collected based on red/green fluorescence intensity of the sperm sample. The raw data is sent to SCSAsoft® for analysis. SCSAsoft® analyzes the raw data and calculates the DNA Fragmentation Index %(DFI); moderately elevated DFI% and high DFI% as well as HDS% [8, 18].

All semen samples were assayed in duplicate with about 5,000 sperm cells in each measurement. Prior to using the flow cytometer alignment is determined using standard fluorescent beads. An AO buffer must pass through the instrument lines for at least 15 min prior to establishing settings with reference samples. The reference sample is chosen for heterogeneity of DNA integrity (eg. DFI% of around 15%) and are diluted to 1–2 Million sperm/mL for use. Clinical Laboratory Improvement Ammendments (CLIA) certification, which ensures quality laboratory testing in the USA, also requires reference samples with low and high DFI% be run for improved quality of analysis. During the use of the SCSA a fresh reference sample is run every 5 to 10 subject samples to exclude drift.

### Blood

Blood was collected in the non-fasting state by standard venipuncture technique into a serum separator test tube, immediately centrifuged and the serum collected. The serum 25-OH Vitamin D level was assayed using a chemiluminescent microparticle immunoassay (Architect, Abbott, Longford, Ireland). This assay was standardized using National Institute of Standards and Technology Standard Reference Material 2971. The limit of quantification with ≤ 20% Coefficient of Variation (CV) was 2.4 ng/mL, with a correlation coefficient of r = 0.99 (95% CI: 0.99,0.99) for liquid chromatography-tandem mass spectrometry. The CV for within-run and between-run assays for low control (20 ng/mL) was 2.2–2.4% and 2.7–3.6%, medium control (40 ng/mL) was 1.8–2.1% and 2.6–3.2%, and high control (75 ng/mL) was 2.2–2.8% and 2.4–4.1%, respectively. Vitamin D deficiency was defined as 25-OH Vitamin D ≤ 20 ng/mL, insufficiency 21–29 ng/mL, and sufficiency as ≥ 30 ng/mL [19].

### Statistics

Sample size determination. Previous studies have demonstrated a statistically significant 8% reduction in sperm motility in subjects with low Vitamin D levels [20]. An alpha of 0.05 and a power of 80% was used to calculate ‘n’. The sample size was determined to be 79 in the Vitamin D deficient group and 79 in the Vitamin D sufficient group. A previous study in this region demonstrated up to 75% Vitamin D deficiency in healthy working, South Dakota males, so the ‘n’ was adjusted to 105 in both groups [21]. Estimating that 10% would decline the SCSA® the ‘n’ was increased to 115 in each group for a total of 230.

An interim analysis was obtained after 111 subjects were recruited and their data were imported into the statistical program, SAS V 9.5. The relationship between the dichotomous variables: alcohol use, tobacco use, and BMI were examined using a chi-square test of independence. The relationship between deficient, insufficient and sufficient Vitamin D levels and sperm parameters were analyzed using an analysis of variance. Correlations between 25-OH-Vitamin D as a continuous variable and semen variables were examined using Pearson correlations. P < 0.05 was considered significant. The interim analysis demonstrated no difference in primary or secondary endpoints. A repeat sample size determination using the largest difference in DFI values from the interim analysis revealed the need for a tenfold increase in each group (*n* = 1150 in each group) to achieve a power of 80% and an alpha of 0.05.

## Results

Of the 111 subjects recruited, exclusions included 3 with no SCSA performed: one azoospermia, one severe oligozoospermia, and one declined SCSA and six with no Vitamin D determination. Of the remaining 102 subjects, 52 had primary and 50 had secondary infertility. Forty (39%) had male factor infertility of which 30 had teratozoospermia, 5 oligoteratozoospermia, 2 asthenozoospermia, 2 oligoasthenoteratozoospermia and 1 with oligospermia. The remaining 61% had normal semen analysis. Vitamin D levels in the 102 subjects demonstrated 24 had deficient Vitamin D (15.28 ± 1.23 ng/mL (mean ± 95% CI)), 43 insufficient Vitamin D (24.87 ± 0.92 ng/mL), and 35 sufficient Vitamin D levels (42.27 ± 5.01 ng/mL). Three subjects had no BMI recorded, and the average BMI of 29.5 was imputed for these cases. The average age was 31.5 with a range of 23 to 50 years and BMI ranged from 18.3 to 52.4 kg/m^2^, with increasing BMI significantly associated with lower Vitamin D levels (Table 1).

**Table 1:** Demographic characteristics of male patients based on Vitamin D levels in Infertile couples

		**Total (*n* = 102)**	**Deficient Vitamin D (≤ 20 ng/mL)** **(*N* = 24)**	**Insufficient Vitamin D (21–29 ng/mL)** **(*N* = 43)**	**Sufficient** **Vitamin D (≥ 30 ng/mL)** **(*N* = 35)**
**Mean Age(range)**		31.5 (23–50)	32.3 (26–47)	31.4 (23–45)	31.1 (23–50)
**BMI kg/m**^**2**^ **(range)**		30.5 (18.3–52.4)	33.9 (22.8–52.4)*	31.2 (20.8–51.2)*	27.3 (18.3–38.2)
**Race(number(%))**	**Caucasian**	92 (90.2)	19 (18.6)	42 (41.2)	31 (30.4)
	**African American**	2 (1.9)	1 (.98)	1 (.98)	0 (0)
	**American Indian**	5 (4.9)	2 (1.9)	0 (0)	3 (2.9)
	**Other**	3 (2.9)	2 (1.9)	0 (0)	1 (.98)
**Tobacco Use**^a,**^ **(number(%))**		22 (21.6)	7 (6.9)	10 (9.8)	5 (4.9)
Alcohol Use^**^ **(number(%))**		88 (86.3)	19 (18.6)	39 (38.2)	30 (29.4)
**Occupation**^b^ **(number(%))**	**Outside**	32 (31.4)	3 (2.9)	15 (14.7)	14 (13.7)
	**Inside**	70 (68.6)	21 (20.6)	28 (27.5)	21 (20.6)

^a^using smokeless or smoking tobacco

^b^job primarily inside or outside

^*^p = 0.0012. Deficient and Insufficient Vitamin D were significantly different when compared to Sufficient levels

^**^Not significant

There was no significant difference in our primary endpoint of Total DFI% and Vitamin D levels; deficient, insufficient, and sufficient. There were also no differences between Vitamin D sufficiency and our secondary outcomes including moderate DFI%, High DFI%, HDS%, sperm density, motility, and morphology (Table 2). Also using the Institute of Medicine’s more restrictive definition of Vitamin D deficiency (Deficient < 13 ng/mL) there was also no significant difference in our primary or secondary endpoints (data not shown).

**Table 2:** Comparison of Vitamin D levels and Sperm parameters

Semen Variable	DeficientVitamin D (< 20 ng/mL) (*N* = 24) Mean(± 95% CI)	Insufficient Vitamin D (21–29 ng/mL) (*N *= 43) Mean (± 95% CI)	Sufficient Vitamin D (≥ 30 ng/mL) (*N *= 35) Mean (± 95% CI)	p*	p**
**Total DFI%**	10(7,13)	10(9.8,12)	11%(8.8,13)	0.7148	0.4561
**Moderate DFI%**	6(4,8)	6(5.8,7.8)	6(4.9,7.1)	0.9764	0.9976
**High DFI%**	3(1.6,4.6)	4(3,5)	5(3.6,6.4)	0.1503	0.0982
**HDS%**	8(6,10)	9(7,11)	9(7.2,11)	0.6776	0.6961
**Sperm Density (Millions/mL)**	86.5(47.7,125.4)	93.7(68.6,118.8)	75.63(54.8,96.5)	0.5969	0.5981
**Motility (%)**	65(59.1,70.9)	65(62,68)	64(60,68)	0.9181	0.8522
**Morphology (%)**	4(3.2,4.8)	5(4.4,6.6)	6(3.3,8.7)	0.6023	0.3818

^*^There was no statistical difference between deficient, insufficient and sufficient Vitamin D levels and any of the Semen and sperm variables

^**^ Combining deficient and insufficient Vitamin D compared to sufficient Vitamin D demonstrated no statistical significant difference

DFI% = DNA Fragmentation Index %; HDS% = High DNA Stainability %

Vitamin D levels varied across the seasons as one would expect with varied sun exposure with the highest levels in Summer (30.45 ± 4.10 ng/mL,mean ± 95% confidence interval) and Fall (32.14 ± 6.29 ng/mL), and lowest in Winter (27.86 ± 5.65 ng/mL) and Spring (26.24 ± 5.48 ng/mL). However, these seasonal differences in Vitamin D did not reach statistical significance. Tobacco consumption was not associated with changes in Vitamin D levels, but abstinence from alcohol resulted in a significant increase in HDS, from 8 ± 0.92% to 12 ± 3.56% (mean ± 95% confidence interval; *p* = 0.0042).

## Discussion

In this prospective, cohort study in males with deficient or insufficient circulating Vitamin D levels there was no difference detected in sperm DFI% compared to those with sufficient Vitamin D. There was also no correlation between Vitamin D levels and HDS%, as well as other semen parameters determined by the routine semen analysis. Results confirmed previous studies which demonstrated decreasing Vitamin D levels with increasing obesity [22, 23]. This study did not demonstrate significant seasonal differences in Vitamin D levels, possibly because of the number of individuals with primarily indoor occupations, which could decrease seasonal variability in sun exposure [22–24].

The role of Vitamin D in the male reproductive system remains controversial with inconsistent results from both animal and human studies. In murine models, hypovitaminosis D has been associated with low circulating testosterone levels, through an indirect genomic effect on calcium metabolism in the Leydig cell affecting testosterone synthesis [25]. In Vitamin D deficient animals circulating Vitamin D levels are positively associated with expression and activity of aromatase, impacting estrogen metabolism. Vitamin D deficient rats with hypocalcemia have impaired spermatogenesis with decreased pregnancy rates, which improved with normalization of calcium levels suggesting that Vitamin D’s role in the reproductive tract is through calcium metabolism [12]. Vitamin D deficient rats have also been discovered to have an elevated DFI% [26]. Our study in the human demonstrated no difference in sperm DFI%, HDS% or other routine semen analysis parameters with varying Vitamin D levels.

In the majority of human studies, Vitamin D deficiency has been associated with increased Sex Hormone Binding Globulin, but no change in circulating testosterone, resulting in a net decrease in biologically active, free testosterone [26]. Vitamin D could impact sperm quality by lowering biologically active testosterone and through non-genomic effects on intracellular calcium and lipid metabolism. In some studies, inadequate Vitamin D has been associated with significant impairment in sperm motility, capacitation, and the acrosome reaction [16, 27, 28]. Similar to our study, other studies have not demonstrated significant changes in sperm parameters with deficient Vitamin D. In particular, one case series described four fertile males with 1,25-dihyroxyvitamin D-resistant rickets due to a non-functioning VDR. These four males had normal serum calcium and phosphorous levels in adulthood, normal semen parameters, and 15 pregnancies resulting in 9 healthy children [29]. Our results combined with previous studies and the knowledge of the integral role of calcium homeostasis in human physiology supports the presence of redundant or local systems to maintain adequate calcium in the male reproductive system [10]. Similar to testosterone metabolism, the presence of Vitamin D metabolizing enzymes in the male reproductive tract could result in higher local levels compared to circulating levels, maintaining normal homeostatic levels during hypovitaminosis. Jueraitetibaike K et al. previously demonstrated no association between seminal and serum Vitamin D levels, supporting different levels within the testicles to enhance spermatogenesis [30].

Limitations of this study include a small sample size. A power analysis using the extreme results from our interim analysis demonstrated a need for a tenfold increase in each group to have a power of 80% and alpha of 0.05. Thus, this study was underpowered to detect a statistical difference. Another limitation is using circulating Vitamin D levels as a surrogate for intratesticular levels. Further studies evaluating seminal plasma levels of Vitamin D could enhance these results [30]. This study is also limited by performance in a single, mid-size Reproductive Endocrinology and Infertility clinic.

## Conclusion

This prospective cohort study demonstrated that human sperm DFI% was not associated with circulating Vitamin D levels; deficient, insufficient, and sufficient. This study also demonstrated no association between circulating Vitamin D levels and HDS% as well as classic sperm parameters as measured by routine semen analysis. This lack of association between these Vitamin D categories and sperm parameters may be related to insufficient difference in levels between deficient, insufficient and sufficient. To further investigate if a more dramatic difference between Vitamin D levels would demonstrate a difference in sperm parameters we used the Institute of Medicine’s more restrictive definition of Vitamin D deficiency (< 13 ng/mL) and there was no statistically difference. Further studies evaluating Vitamin D levels locally in the male reproductive tract are needed to further evaluate the role Vitamin D may play in male reproduction.

## Data Availability

The datasets generated and analyzed during the current study will be available at ClinicalTrials.gov.

## Abbreviations

AO: Acridine Orange
BMI: Body Mass Index
CI: Confidence Interval
CV: Coefficient of Variation
DFI%: DNA Fragmentation Index%
DNA: Deoxyribonucleic acid
EMR: Electronic Medical Record
EDTA: Ethylenediaminetetraacetic acid
HDS%: High DNA Stainability %
ICSI: Intracytoplasmic Sperm Injection
IVF: In Vitro Fertilization
mL: Milliliters
mW: Milli-Watts
nm: Nanometers
ng: Nanograms
n: Sample size
Na2HPO_4_: Disodium Phosphate
NaCl: Sodium Chloride
SCSA: Sperm Chromatin Structure Assay
TUNEL: Terminal deoxynucleotidyl transferase dUTP nick end labeling
VDR: Vitamin D Receptor
WHO: World Health Organization

## Declarations

### Previous Presentation

This study was presented at the Proceedings of the American Society of Andrology Annual Conference 2021; 2021 Apr 11–14; teleconference.

This study was approved by the Institutional Review Board at Sanford Health (IRB00001575) and was registered with Clinical Trials.gov (MOD00002311) prior to recruiting patients. All patients received oral and written consent prior to participation.

## References

[1] Agarwal A, Baskaran S, Parekb N, Cho CL, Henkel R, Vij S (2021). Male infertility. Lancet.

[2] Salim Abu Naser S, Ibrahim Alhabbash M. Male Infertility Expert System Diagnoses and Treatment. Am J innov res appl sci. 2016;2(4):181–92.

[3] De Geyter C. European pregnancy rates from IVF and ICSI “appear to have reached a peak.” ESHRE. https://www.eshre.eu/Annual-Meeting/Vienna-2019/Media/2019-Press-releases/EIM Accessed January 5, 2021.

[4] Hamada A, Esteves SC, Nizza M, Agarwal A (2012). Unexplained Male infertility: diagnosis and Management. Int Braz J Urol.

[5] Lewis SEM (2007). Is sperm evaluation useful in predicting human fertility?. Reproduction.

[6] Ribas-Maynou J (2013). Comprehensive analysis of sperm DNA fragmentation by five different assays: TUNEL assay, SCSA, SCD test and alkaline and neutral Comet assay. Andrology.

[7] Fernández JL (2003). The Sperm Chromatin Dispersion Test: A Simple Method for the Determination of Sperm DNA Fragmentation. J Androl.

[8] Evenson DP (2016). The Sperm Chromatin Structure Assay (SCSA(®)) and other sperm DNA fragmentation tests for evaluation of sperm nuclear DNA integrity as related to fertility. Anim Reprod Sci.

[9] Evenson, DP. Sperm Chromatin Structure Assay (SCSA®): 30 years of experience with the SCSA®. In:Zini A, Agarwal A. eds. Sperm Chromatin: Biological and Clinical Applications in Male Infertility and Assisted Reproduction.New York; Springer;2011p125–49.

[10] Haussler MR, Jurutka PW, Mizwicki M, Norman AW (2011). Vitamin D receptor (VDR)-mediated actions of 1α, 25(OH)_2_vitamin D_3_: genomic and non-genomic mechanisms. Best Pract Res Clin Endocrinol Metab.

[11] Hansen LB, Lorenzen M, Bentin-Ley U, Nielsen JE, Krog H, Berg AH (2019). Presence of the vitamin D inactivating enzyme CYP24A1 in human sperm and prediction of the success of intrauterine insemination: A prospective study. J Steroid Biochem Mol Biol.

[12] Zanatta L, Zamoner A, Zanatta AP, Bouraїma-Lelong H, Delalande C, Bois C (2011). Nongenomic and genomic effects of 1α,25(OH)2 vitamin D3 in rat testis. Life Sci.

[13] Cito G, Cocci A, Micelli E, Gabutti A, Russo GI, Coccia ME (2020). Vitamin D and Male Fertility: An Updated Review. World J Mens Health.

[14] Blomberg Jensen M, Bjerrum PJ, Jessen TE, Nielsen JE, Joensen UN, Olesen IA (2011). Vitamin D is positively associated with sperm motility and increases intracellular calcium in human spermatozoa. Hum Reprod.

[15] Hammoud AO, Wayne Meikle A, Matthew Peterson C, Stanford J, Gibson M, Carrell DT (2012). Association of 25-hydroxy-vitamin D levels with semen and hormonal parameters. Asian J Androl.

[16] Blomberg Jensen M, Jørgensen A, Nielsen JE, Bjerrum PJ, Skalkam M, Petersen JH (2012). Expression of the vitamin D metabolizing enzyme CYP24A1 at the annulus of human spermatozoa may serve as a novel marker of semen quality. Int J Androl.

[17] Cooper TG, Noonan E, von Eckardstein S, Auger J, Baker HWG, Behre HM (2010). World Health Organization reference values for human semen characteristics. Hum Reprod Update.

[18] Evenson DP (2012). Sperm Chromatin Structure Assay (SCSA®). Methods Mol Biol.

[19] Holick MF, Binkley NC, Bischoff-Ferrari HA, Gordon CM, Hanley DA, Heaney RP (2011). Evaluation, treatment, and prevention of vitamin D deficiency: an Endocrine Society clinical practice guideline. J Clin Endocrinol Metab.

[20] Blomberg Jensen MB, Lawaetz JG, Andersson AM, Petersen JH, Nordkap L, Bang AK (2016). Vitamin D deficiency and low ionized calcium are linked with semen quality and sex steroid levels in infertile men. Hum Reprod.

[21] Huntington MK, Shafer CW, Pudwill R, Boer L, Kendall J (2010). Prevalence of vitamin D deficiency among immigrants to South Dakota. S D Med.

[22] Konradsen S, Ag H, Lindberg F, Hexeberg S, Jorde R (2008). Serum 1,25-dihydroxy vitamin D is inversely associated with body mass index. Eur J Nutr.

[23] Wortsman J, Matsuoka LY, Chen TC, Lu Z, Holick MF (2000). Decreased bioavailability of vitamin D in obesity. Am J Clin Nutr.

[24] Kasahara AK, Singh RJ, Noymer A (2013). Vitamin D (25OHD) Serum Seasonality in the United States. PLoS ONE.

[25] Sonnenberg J, Luine VN, Krey LC, Christakos S (1986). 1,25-Dihydroxyvitamin D3 treatment results in increased choline acetyltransferase activity in specific brain nuclei. Endocrinology.

[26] Merino O, Sánchez R, Gregorio BM, Sampaio FJ, Risopatrón J (2018). Effects of Diet-Induced Obesity and Deficient in Vitamin D on Spermatozoa Function and DNA Integrity in Sprague-Dawley Rats. Biomed Res Int.

[27] Yang B, Sun H, Wan Y, Wang H, Qin W, Yang L (2012). Associations between testosterone, bone mineral density, vitamin D and semen quality in fertile and infertile Chinese men. Int J Androl.

[28] Blomberg Jensen M, Dissing S (2012). Non-genomic effects of vitamin D in human spermatozoa. Steroids.

[29] Tiosano D, Weisman Y (2019). Reproductive history of patients with hereditary 1,25-dihydroxyvitamin D-resistant rickets. Fertil Steril.

[30] Jueraitetibaike K, Ding Z, Wang DD, Peng LP, Jing J, Chen L (2019). The effect of vitamin D on sperm motility and the underlying mechanism. Asian J Androl.

